# Patients’ perceptions of palliative care: adaptation of the Quality from the Patient’s Perspective instrument for use in palliative care, and description of patients’ perceptions of care received

**DOI:** 10.1186/s12904-015-0049-4

**Published:** 2015-11-02

**Authors:** Tuva Sandsdalen, Ingrid Rystedt, Vigdis Abrahamsen Grøndahl, Reidun Hov, Sevald Høye, Bodil Wilde-Larsson

**Affiliations:** Department of Health Studies, Faculty of Public Health, Hedmark University College, Postbox 400, 2418 Elverum, Norway; Department of Health Science, Faculty of Health, Science and Technology, Discipline of Nursing Science, Karlstad University, 651 88 Karlstad, Sweden; Faculty of Health and Social Studies, Østfold University College, Postbox 700, 1757 Halden, Norway

**Keywords:** Palliative care, Quality of healthcare, Instrument development, Factor analysis, Statistical, Patient perspectives

## Abstract

**Background:**

Instruments specific to palliative care tend to measure care quality from relative perspectives or have insufficient theoretical foundation. The instrument Quality from the Patient’s Perspective (QPP) is based on a model for care quality derived from patients’ perceptions of care, although it has not been psychometrically evaluated for use in palliative care. The aim of this study was to adapt the QPP for use in palliative care contexts, and to describe patients’ perceptions of the care quality in terms of the subjective importance of the care aspects and the perceptions of the care received.

**Method:**

A cross-sectional study was conducted between November 2013 and December 2014 which included 191 patients (73 % response rate) in late palliative phase at hospice inpatient units, hospice day-care units, wards in nursing homes that specialized in palliative care and homecare districts, all in Norway. An explorative factor analysis using principal component analysis, including data from 184 patients, was performed for psychometric evaluation. Internal consistency was assessed by Cronbach’s alpha and paired *t*-tests were used to describe patients’ perceptions of their care.

**Results:**

The QPP instrument was adapted for palliative care in four steps: (1) selecting items from the QPP, (2) modifying items and (3) constructing new items to the palliative care setting, and (4) a pilot evaluation. QPP instrument specific to palliative care (QPP-PC) consists of 51 items and 12 factors with an eigenvalue ≥1.0, and showed a stable factor solution that explained 68.25 % of the total variance. The reliability coefficients were acceptable for most factors (0.79–0.96). Patients scored most aspects of care related to both subjective importance and actual care received as high. Areas for improvement were symptom relief, participation, continuity, and planning and cooperation.

**Conclusion:**

The QPP-PC is based on a theoretical model of quality of care, and has its roots in patients’ perspectives. The instrument was developed and psychometrically evaluated in a sample of Norwegian patients with various diagnoses receiving palliative care in different care contexts. The evaluation of the QPP-PC shows promising results, although it needs to be further validated and tested in other contexts and countries.

## Background

Patients with life-threatening illnesses and in need of palliative care may have cancer or non-cancer illnesses [1]. The structure of palliative care for these patients may differ between countries [2]. In Norway they receive help from a public healthcare system that has specialized and non-specialized services in palliative care [2], in either community care or specialist healthcare contexts [3]. Community care serves patients at home, via home care or their general practitioner (GP), and patients in nursing homes. Specialist healthcare serves patients in hospitals and specialist services. Hospice care may be part of community care or specialist healthcare [4], and offers specialized inpatient care and day-care services.

The need to have knowledge and evaluate the quality of palliative care is recognized both internationally and in Norway [4–6]. Patients’ perceptions of their care may be seen as one aspect of quality of care [7, 8] and is considered important for development and improvement of palliative care [5, 6, 8–11]. To gain such knowledge, validated instruments are needed, which should be tested in different settings and on patients with different illnesses [12, 13].

Previous instruments have measured quality in palliative care, often from the perspectives of the relatives, e.g. FAMCARE [14], Views Of Informal Carers – Evaluation of Services (VOICES) [15] and Care Evaluation Scale (CES) [16]. Although some of these instruments have been modified and used to measure patients’ perspectives of care [17–19], instruments derived from patients’ perspectives are available, e.g. the Canadian Health Care Evaluation Project (CANHELP) that measure satisfaction of various aspects of palliative care [20], the Quality of end-of-life care (QEOLC) [21], and the Quality of end-of-life care and satisfaction with treatment quality-of-care scale (QUEST) [22]. The last two measure parts of palliative care; physicians’ skills and interaction with healthcare personnel.

The advantage of using existing instruments to measure patients’ perspectives of palliative care quality is that they have been developed within a palliative care context, including patients’ views in the development process. The importance of instruments with a foundation of a theoretical model of care quality that is based on patients’ perspectives and conceptions of the area can be found in the literature [23], and ensures the measurement of all important aspects of care quality from the patients’ perspectives. However, there is a lack of instruments explicitly founded on a theoretical model of care quality from patients’ perspectives.

The Quality from the Patient’s Perspective (QPP) instrument is based on a theoretical model of quality of care from the patient’s perspective [24]. It was developed from interviews with patients using a grounded theory approach, and the instrument was psychometrically tested using factor analysis [25], and further validated by a dimensional analysis of all items using structural equation modelling [26]. A short version of the QPP has been validated [27], frequently used and psychometrically tested in several contexts [28, 29]. In addition, a modified version of the short version has been developed for advanced home care, a palliative homecare service connected to a palliative medicine unit, which provides qualified medical care in the context of the patients’ homes [30].

From this model, quality of care can be understood in the light of two conditions: the resource structure of the care organization, consisting of person-related, physical and administrative environmental qualities, and patients’ preferences, consisting of human and rational aspects [24]. In this framework, the QPP instrument measures patients’ perceptions of quality of care via items related to four dimensions: the medical–technical competence of the care givers, the degree of identity orientation in the attitudes and activities of the caregivers, the physical–technical conditions of the care organization and its sociocultural atmosphere [24]. Patients evaluate the quality of care in two ways: how they perceive the reality of the quality of care (PR) and the subjective importance of the various aspects of care (SI).

Although the QPP is based on a theoretical model derived from patients’ perspectives and was previously modified and used in palliative home care [30, 31], it has not undergone a psychometric evaluation in palliative care, and does not adequately address all aspects identified as important to patients receiving palliative care [32]. Further development and psychometric evaluation of QPP for a palliative care context are needed.

The aim of this study was to: (1) adapt the QPP for use in palliative care contexts, and (2) describe patients’ perceptions of the care quality in terms of the subjective importance of the care aspects and the perceptions of the care received.

## Methods

### Settings and participants

Data for this cross-sectional study were collected between November 2013 and December 2014, in two hospice inpatient units (H), two hospice day care units (HD), two wards in nursing homes that specialized in palliative care (NH) and two homecare districts (HC), all in Norway. Six of the settings were specialized (H, HD and NH) and two were non-specialized in palliative care (HC). The settings represented rural and urban locations in the eastern part of Norway.

Patients were consecutively recruited when they met the following inclusion criteria: adult, no cognitive impairment (which was judged by the responsible registered nurse [RN]), understand Norwegian, receive assistance from the services for at least 3 days, have malignant or non-malignant advanced, life-threatening illness in a late palliative phase (as judged by the responsible RN) and guided by a nurse’s negative response to the question: ‘Would you be surprised if this patient dies within the next year?’ [33] (this was judged by the responsible RN). Eligible patients should be included in a palliative care service or, if not, there should be documentation in the patients’ charts indicating that they were in a late palliative phase. The patients’ physicians and one of the researchers (TS) were consulted when uncertainties arose about the inclusion criteria. In addition, eligible patients should be personally aware that they were in a palliative phase (having a life-threatening illness) and that they received palliative care (as judged by the responsible RN).

The instrument was delivered to 262 patients and 191 participated by returning it (response rate = 73 %). Those who did not respond (*n* = 71) did not differ significantly from the responding group with regard to age (*P* = 0.569) or gender (*P* = 0.117).

### Measures

QPP is an instrument developed to measure the quality of care from the patients’ perspectives [25–27]. In this study, a QPP instrument specific to palliative care (QPP-PC) was adapted to measure quality of care from the perspectives of patients with different life-threatening illnesses in diverse palliative care settings. Modifications of the QPP items and development of new items were mainly based on a review of the literature [32] and of symptoms presented in the revised version of the Edmonton Symptom Assessment System (ESAS-r) [34, 35], in addition to the research team’s expert knowledge in the field, based on experience working with patients in palliative phase and by conducting research in the context of palliative care. The process for the adaptation of the QPP instrument to palliative care is presented in the result section.

#### Administration of the QPP-PC to patients

Administration of the QPP-PC to the patients resulted in 69 items making up the QPP dimensions: medical–technical competence (11 items); identity-oriented approach (20 items); physical–technical conditions (2 items); sociocultural atmosphere (8 items); and, in addition, context-specific items (28 items). Patients answered each item in two ways. First, patients scored their opinions of the quality of actual care received, perceived reality (PR), related to the sentence ‘This is what I experience …’ (e.g. personnel are respectful to me). Then patients scored the subjective importance of care aspects (SI), related to the sentence ‘This is how important this is to me …’ (e.g. personnel are respectful to me). A four-point Likert scale, ranging from 1 (do not agree at all) to 4 (fully agree), was used for PR, and for SI from 1 (of little or no importance) to 4 (of the very highest importance). A non-applicable alternative was available for both responses.

In addition, the questionnaire consisted of 10 background questions.

### Procedure and data collection

Patients were asked to participate by the RN who was responsible for each ward and for screening patients. This nurse provided written and oral information about the study; this information explained that participation was voluntary and that patients’ answers were treated confidentially. Patients gave oral and written consent to participate in the study. Patients were offered help from one of the researchers (TS) to complete the questionnaire, with 50 patients receiving such help. The participants were told that they could use as much time as they needed to complete the questionnaire and return it in a sealed envelope.

### Ethical approval

The Regional Committee for Medical and Health Research Ethics in South-East Norway (REC) considered that the study could be conducted without any further approval from them (REC no. 2013/865). Therefore the study was reported to the Norwegian Social Science Data Services (NSD no. 34770). Participation of the settings was approved by the head administrators. Permission to use the QPP was obtained from the developer.

### Statistical analysis

Data were analysed using IBM SPSS Statistics, version 22. Seven patients were excluded from the statistical analysis due to the proportion of items answered ‘not applicable’ (or missed observations) only made it possible to calculate less than six out of 12 factors (SI and PR) or one out of four dimensions (SI and PR), leaving 184 patients left for statistical analysis. Descriptive statistics were used to examine patients’ characteristics. Paired sample *t*-tests were used to investigate patients’ perceptions of the care in terms of differences in subjective importance (SI) and perceived reality (PR) [36]. An independent *t*-test or Pearson *χ*^2^ test was used for drop-out analysis, as appropriate [36].

Exploratory factor analysis (EFA) using principal component analysis (PCA) was chosen for exploration of the underlying structure among the items [36], because the QPP instrument was modified for use in a new context with no assumption of a known hypothesis about the dimensionality of a set of items [36, 37] . Before PCA, the data’s suitability for factor analysis (EFA) was assessed using Bartlett’s test of sphericity, which tests the overall significant differences in the correlation matrix, and the Kaiser–Meyer–Olkin (KMO) test to check that the sample adequacy was appropriate [36, 37] . To guide the extraction of factors, Kaiser’s criterion was used and factors with an eigenvalue ≥1.0 were obtained for further analysis [36]. To give further support to the extraction of factors, varimax rotation methods with Kaiser normalization were used, which assume no correlation of the underlying factors [36], to ascertain how the items correlate with the different components of the quality-of-care construct. Items loading at ≥0.4 were considered as acceptable loadings to the factor [37], and the items were included in the factor where they loaded the highest. Labelling of the factors was based on the content of the items correlating with the factors [37].

Based on the theoretical model underlying the QPP instrument developed from patients’ perceptions of quality of care, a PCA was conducted for SI and PR scales. Ratings from the SI scale are presented since these scores were considered to reflect general values compared with perceived reality scores, which reflect specific conditions of the settings [25].

The reliability was assessed using Cronbach’s α. Cronbach’s α analyses were carried out on dimensions and factor levels of both subscales (PR and SI) and values >0.7 were regarded as desirable [37].

Since QPP-PC was developed to measure the care quality in a number of different settings and for patients with several different illnesses, “not applicable” responses will always be present. For the QPP-PC dimensions and factors, a mean value was calculated based on the individual participant’s response to the remaining items in the respective dimension or factor. These mean values were used in the paired *t*-tests.

For the PCA, when participants had responded ‘not applicable’ to an item, a mean of the remaining responses in the respective variable substituted the ‘not applicable’ response [38]. Since the ‘not applicable’ response is coded together with missing observations, any missing observation was also substituted by a mean of the remaining responses in the respective variable. The statistical significance was assumed at *P*-level <0.05.

## Results

### The adaptation of QPP to palliative care

The adaptation of the QPP instrument to be specific to palliative care was performed in four steps.

#### First step: selection of existing items

This step comprised selection of QPP items relevant to the palliative care context. They were selected from the short version of the QPP instrument [27, 39] (20 items), the full QPP version (8 items) [25, 26] and a QPP previously modified for advanced home care [30] (personal communications with the author Wilde-Larsson) (7 items), comprising a total of 35 items. Of these, 25 items were taken verbatim or were slightly abbreviated, e.g. the item ‘I receive the best physical care, e.g. help to take care of my personal hygiene’ was abbreviated to ‘I receive the best possible help to take care of my personal hygiene’.

#### Second step: modification of existing items

Of the 35 original QPP items, 10 were modified to suit the palliative care context better by altering wording, e.g. the item ‘Feeling that the nurses showed interest in my outlook on life [my spiritual needs]’ was modified to ‘The nurses support me in tending to my spiritual and existential needs’. In addition, QPP items about nurses and doctors were modified by adding items to cover multiprofessional staff (labelled ‘other personnel’). This comprised 8 additional items, with a total of 43 items.

#### Third step: construction of new items

Some 34 new items were then developed to cover palliative-specific aspects of care that were not or were only partly covered by the QPP. These new items included: symptom management of tiredness and depression (2 items); preservation of dignity (3 items); financial concerns (1 item); information about diagnosis and prognosis (2 items); choosing place of care (1 item); practical support (1 item); sufficiency of multiprofessional care (10 items); support, information and participation of the family (4 items); a secure atmosphere (1 item); support from fellow patients (1 item); and continuity, planning and cooperation (8 items). The total number of items at this stage was 77.

#### Fourth step: pilot evaluation

A pilot evaluation of the face validity, content validity and comprehensibility of the items, and clarity of instructions, was conducted by the researcher (TS). This involved interviews with eight patients in late palliative phases after completion of the questionnaire. Patients in the pilot study were recruited from two hospice inpatient units (*n* = 4) and two hospice day-care units (*n* = 4). Four men and four women, with ages ranging from 45 to 70 years, participated. The interview consisted of the following topics: relevance of items to quality of palliative care, perceptions about completing the questionnaire, length of the questionnaire, comprehensibility of the items and instructions for filling out the questionnaire, and suggestions for improving items.

The results from the pilot evaluation showed that items comprised quality of palliative care, and that items and instructions were comprehensible, but the number of items was perceived as too high. Based on these results, critical assessment of the items was performed by the research group, resulting in a reduction in the wording of 31 items. In addition, a conflation of items was performed which resulted in the removal of eight items considered to be covered by other items. For example, the two items measuring a secure atmosphere and a pleasant atmosphere were reduced to: ‘There is a pleasant and secure atmosphere at the ward’. This step resulted in a total of 69 items.

### Patient characteristics

Age ranged from 41 to 94 years, and 50 % of the participants were aged >67 years (*n* = 184). Of the participants 56 % were women and 52 % lived alone. Most had Norwegian as their first language (93 %), with 76 % having a cancer diagnosis and 70 % just one diagnosis. With regard to education, 24.6 % had education up to primary school, 38.5 % to high school and 36.9 % to university education (Table 1).

**Table 1:** Patient characteristics (*n* = 184)

	*n* (%)	Missing
Age (years)		7
Mean age (SD), range	66.51 (11.71), 41–94	
Gender		3
Female	101 (55.8)	
Male	80 (44.2)	
Education		5
Primary school or equivalent	44 (24.6)	
High school or equivalent	69 (38.5)	
University/university college	66 (36.9)	
First language		1
Norwegian	171 (93.4)	
Sami	0	
Other Nordic language	3 (1.6)	
Other European language	8 (4.4)	
Non-European language	1 (0.5)	
Type of illness		1
Malignant illness (cancer)	139 (76.0)	
Non-malignant illness (e.g. COPD, HF, MS, ALS, Parkinson’s disease)	29 (15.8)	
Mixed malignant and non-malignant illnesses	15 (8.2)	
Number of illnesses		1
One diagnosis	128 (69.9)	
Two or more illnesses	55 (30.1)	
Setting		
Hospice inpatient care	69 (37.5)	
Hospice day care	51 (27.7)	
Palliative care units in nursing homes	29 (15.8)	
Home care	35 (19.0)	
Time in care (days)		11
3–7 days	30 (17.3)	
8–30 days	51 (29.5)	
31–182 days (1–6 months)	47 (27.2)	
> 183 (6 months)	45 (26.0)	
Living conditions		1
Living alone	95 (51.9)	
Living with a partner	69 (37.7)	
Living with children aged <18 years	11 (6.8)	
Living with others	8 (4.4)	
The amount of contact with family or friends		1
Daily	107 (58.5)	
Several times a week	62 (33.9)	
Once a week to once a month	14 (7.7)	
Less than once a month	0	
No contact with family or friends	0	
The sufficiency of contact with family or friends		1
Too often	5 (2.7)	
Sufficient	155 (84.7)	
Too seldom	23 (12.6)	
Religious affiliation		12
No	90 (52.3)	
Yes	82 (47.7)	

For categorical variables *n* (%) is presented. For continuous variables mean (SD) and range are presented

ALS, amyotrophic lateral sclerosis; COPD, chronic obstructive pulmonary disorder; HF, heart failure; MS, multiple sclerosis

### Construct validity measured by EFA using PCA

To ensure a stable factor solution, further reductions of items were needed before doing the PCA. A critical assessment of items was carried out, guided by the results of the descriptive analysis of the response alternative ‘not applicable’ (and missed observations). Of the 69 items, 17 were excluded because a high proportion (>35 %) of patients scored them as ‘not applicable’/missed observations and these items were assessed to also be covered by existing items.

The item about the atmosphere on the ward was ‘not applicable’ to those receiving home care, but was perceived to be important for patients in hospices (day and inpatient care) and nursing homes, so therefore it was kept in the instrument as a single item. This left 51 items for further psychometric evaluation, and one additional single item.

As Bartlett’s test of sphericity was significant (*P* <0.05), this indicated that the data were considered suitable for FA [37]. The KMO value was 0.82, indicating that the sample should produce reliable and distinct factors [36]. The initial PCA analysis showed that the structure of the answers of the two items ‘I receive the best possible help to take care of my personal hygiene’ and ‘I receive the best possible medical care’ produced different patterns from the other items in the factor. However, as the SI scores were high, these two items were kept as single items because of their importance to the patients.

For the remaining 49 items PCA revealed 12 factors with an eigenvalue >1.0, which explained 68.25 % of the total variance. Extracted factors with eigenvalues >1.00 for the SI-scale at item- and factor levels are shown in Table 2.

**Table 2:** Rotation matrix for the 12-factor structure of the 49-item QPP-PC, SI scale (*n* = 184)

Items/Factors	Respect and empathy	Symptom relief	Information	Spiritual and existential	Meaning fullness	Participation	Planning and cooperation	Honesty	Relatives and friends	Exhaustion	Access to help, food and equipment	Continuity
Help for:												
Pain		0.332										
Nausea		0.722										
Lack of appetite		0.525										
Shortness of breath		0.392										
Anxiety		0.762										
Depression		0.763										
Lack of sleep		0.726										
Constipation/diarrhoea		0.575										
Help for:												
Tiredness										0.797		
Drowsiness										0.774		
Waiting time											0.574	
Food and drink											0.484	
Apparatus and equipment											0.522	
Doctors’ understanding	0.664											
Nurses’ understanding	0.698											
Other personnel’s^a^ understanding	0.494											
Doctors’ respect	0.859											
Nurses’ respect	0.837											
Other personnel’s^a^ respect	0.656											
Doctors gives honest answers								0.512				
Nurses give honest answers								0.664				
Other personnel^a^ give honest answers								0.795				
Information care and treatment			0.488									
Information on medication			0.346									
Information on diagnosis			0.652									
Information on prognosis			0.681									
Information on self-care			0.754									
Responsible doctor			0.525									
Responsible nurse			0.516									
Participation in medical care						0.760						
Participation in nursing care						0.767						
Participation in plan for care						0.514						
Participation in place of care						0.424						
Doctors give spiritual support				0.876								
Nurses give spiritual support				0.892								
Other personnel^a^ give spiritual support				0.885								
Doctors support meaningfulness					0.831							
Nurses support meaningfulness					0.785							
Other personnel^a^ support meaningfulness					0.718							
Relatives treated with respect									0.775			
Relatives receiving support									0.789			
Relatives’ participation in decisions about care									0.705			
Same doctor												0.748
Same nurse												0.481
Individualized care												0.400
Planning medical care							0.747					
Planning nursing care							0.773					
Cooperative personnel							0.764					
Cooperative services							0.530					
Number of items	6	8	7	3	3	4	4	3	3	2	3	3
Eigenvalue	12.79	3.74	2.79	2.30	2.15	1.96	1.63	1.52	1.34	1.26	1.15	1.06
Explained variance^b^	26.10	7.63	5.69	4.70	4.39	4.01	3.33	3.10	2.74	2.56	2.35	2.16

Extraction method: principal component analysis; rotation method; varimax with Kaiser normalization

^a^Other personnel: assistant nurses, priest, physiotherapist, occupational therapist or social worker

^b^Explained variance in percentage: total explained variance in percentage = 68.25 % (12 factors)

The factor solution was supported by the varimax rotation matrix, and revealed a number of strong loaded items (>0.4) in each factor. Three items loaded just below 0.4 (‘Help for pain’, ‘Help for shortness of breath’ and ‘Information about medication’). These items were kept because they showed sufficient correlation [37] and because patients scored them as of high to highest importance (SI score: mean 3.07–3.58; standard deviation [SD] 0.68–1.03). All items were kept in the factor in which they loaded the highest, except for the following items: ‘Help for shortness of breath’, ‘Information about medication’ and ‘Cooperative services’, which were placed according to knowledge in the field.

PCA was also carried out for the PR scale. The results from the PCA for the items within the PR scale showed similar patterns, as demonstrated for the SI scale in Table 2.

This was also the case for the results of PCA analysis at the dimensional level for the QPP dimensions: medical–technical competence (MT), identity-oriented approach (ID), physical–technical conditions (PT) and sociocultural atmosphere (SC) for both SI and PR.

### Reliability

Internal consistency estimated with Cronbach’s α is shown in Table 3. At the factor level range, α is between 0.79 and 0.96, except for the factors ‘Access to help, food and equipment’ (α = 0.65) and ‘Continuity’ (α = 0.55). At the dimensional level values ranged between 0.91 and 0.94 for the SI scale, apart from the PT dimension (α = 0.65).

**Table 3:** The QPP-PC, inclusive dimensions, factors, 49 items and 3 single items

Dimensions/Factors/Items	Cronbach’s α^a^	
	Subjective importance (SI)	Perceived reality (PR)
**Medical–technical competence** (12 items)	0.91	0.91
Symptom relief	0.91	0.88
I receive the best possible help for:		
Pain		
Nausea/vomiting		
Loss of appetite		
Shortness of breath		
Depression (feeling sad)		
Anxiety (feeling nervous)		
Lack of sleep		
Constipation/diarrhoea		
Exhaustion	0.89	0.88
I receive the best possible help to take care of my:		
Tiredness (lack of energy)		
Drowsiness (feeling sleepy)		
I receive:		
The best possible medical care (single item)		
The best possible help to take care of my personal hygiene (single item)		
**Physical–technical conditions** (3 items)	0.65	0.44
Access to help, food and equipment	0.65	0.44
I receive help within an acceptable waiting times		
I receive food and drink that I like		
I have access to the necessary equipment		
**Identity-oriented approach** (20 items)	0.94	0.88
Information	0.83	0.83
I receive useful information on:		
How care and treatments will take place		
The effects and use of medicine		
My illness and my symptoms		
What I may expect in the near future (development of the illness and symptoms, my health and function)		
How to take care of myself		
Which doctor are responsible for my medical care		
Which nurse are responsible for my nursing care		
Honesty	0.87	0.78
The personnel seem to give me honest answers to my questions:		
Doctors		
Nurses		
Other personnel		
Respect and empathy	0.92	0.81
The personnel seem to understand how I experience my situation:		
Doctors		
Nurses		
Other personnel		
The personnel are respectful towards me:		
Doctors		
Nurses		
Other personnel		
Participation	0.88	0.81
I have good opportunity to participate in the decisions that apply to:		
Medical care		
Nursing care		
Individual plan for my care		
Choose where to receive my care		
**Socio-cultural atmosphere** (17 items)	0.92	0.90
Meaningfulness	0.96	0.89
The personnel support and assist me in living the rest of my life in a meaningful way:		
Doctors		
Nurses		
Other personnel		
Spiritual and existential	0.97	0.96
The personnel support and assist me in tending to my spiritual and existential needs:		
Doctors		
Nurses		
Other personnel		
Relatives and friends	0.79	0.66
My relatives:		
And friends are treated with respect		
Receive the best possible help, support and care		
May participate in decisions about my care, according to my preferences		
Continuity	0.55	0.53
I usually receive help from the same:		
Doctor		
nurse		
My care is determined by my own requests and needs rather than staff procedures		
Planning and cooperation	0.79	0.83
There is good planning of my:		
Medical care		
Nursing care		
The personnel cooperate well		
All the health and welfare services that I receive are well coordinated		
There is a pleasant and secure atmosphere on the ward (single item)		

^a^Cronbach’s α, at dimensional and factor levels. For dimensional level, single items are included in the α value

Other personnel refers to: assistant nurse, priest, physiotherapist, occupational therapist or social worker

### Patients’ perceptions of quality of palliative care

The highest levels of SI as well as PR were reported for the factors ‘Respect and empathy’ and ‘Honesty’ in the ID dimension. The highest levels for SI and PR were reported for the single item about the atmosphere in the SC dimension. In addition, high levels of SI were reported for the single item medical care in the MT dimension

The lowest levels for SI as well as PR were reported for the factor ‘Exhaustion’ in the MT dimension and ‘Spiritual and existential’ in the SC dimension.

When comparing patients’ scores for the SI and PR scales, SI scales were statistically significantly higher for the factor ‘Symptom relief’ in the MT dimension, for the factors “‘Information’ and ‘Participation’ in the ID dimension and for the factors “‘Continuity’ and ‘Planning and cooperation’ in the SC dimension. In addition, for the single items, the SI scale was statistically significantly higher than the PR scale for the item about medical care (MT dimension) and for the item about atmosphere (SC dimension), the SI scale was significantly lower than the PR scale (Table 4).

**Table 4:** Comparison of patients’ perceptions of subjective importance and care received, by dimensions, factors and single items

Dimensions/factors/single items	Subjective importance (SI)	Perceived reality (PR)		
	Mean (SD)	Mean (SD)	*n*	*p* value
Medical–technical competence	3.16 (0.61)	3.04 (0.70)	180	0.018
Symptom relief	3.22 (0.62)	3.10 (0.72)	180	0.012
Fatigue/exhaustion	3.01 (0.83)	2.88 (0.94)	164	0.092
Medical care (single item)	3.74 (0.53)	3.57 (0.73)	181	<0.01
Personal hygiene (single item)	3.47 (0.69)	3.49 (0.70)	125	0.790
Physical–technical conditions	3.49 (0.55)	3.51 (0.58)	182	0.686
Access to help, food and equipment	3.49 (0.55)	3.51 (0.58)	182	0.686
Identity-oriented approach	3.51 (0.46)	3.36 (0.48)	184	<0.01
Respect and empathy	3.66 (0.50)	3.66 (0.41)	181	0.923
Honesty	3.70 (0.44)	3.74 (0.45)	178	0.291
Information	3.38 (0.57)	3.11 (0.72)	182	<0.01
Participation	3.31 (0.75)	3.11 (0.83)	176	<0.01
Sociocultural atmosphere	3.39 (0.49)	3.34 (0.52)	183	0.120
Spiritual and existential	2.98 (1.04)	2.99 (0.94)	122	0.837
Meaningfulness	3.49 (0.71)	3.47 (0.68)	180	0.688
Relatives and friends	3.61 (0.51)	3.59 (0.55)	153	0.545
Continuity	3.27 (0.66)	3.16 (0.71)	180	0.043
Planning and cooperation	3.57 (0.55)	3.45 (0.64)	171	<0.01
Atmosphere (single item)	3.79 (0.43)	3.89 (0.33)	123	<0.01

*p* values refer to differences in paired sample *t*-tests. A statistical significance was assumed at *p*-level <0.05

## Discussion

### Methodological considerations

One way of assessing whether the QPP-PC is a reliable and valid instrument is to measure the QPP-PC according to the criteria stated by van Campen et al. [23]: instruments should (1) be based on a theoretical foundation, (2) contain a subscale representing different aspects of quality of care, (3) be tested for reliability and validity, and (4) be feasible in large populations. In our opinion, the QPP-PC meets these criteria.

The items in the QPP-PC still reflect all four dimensions of the theoretical model of quality of care derived from patients’ perspectives, on which the QPP is based. Sitzia[40] elaborated on the validity assessment of instruments by stating that studies should provide results for content validity comprising strategies for item generation and content testing. Items in the QPP-PC were developed based on the perspective of patients receiving palliative care, ensuring that the items developed are perceived as relevant for these patients. The pilot test showed good face and content validity; this was supported by high patients’ scores on the SI scale for each dimension, factor and single item (Table 4), which was also the case at the level of the items (not reported). Consequently, this supported the content validity of the instrument [40].

The construct validity [40] was evaluated by performing an explorative factor analysis using PCA, which showed a stable 12-factor solution for the SI and PR scales, with most items correlating strongly with the factors. Compared with the original QPP, three new factors appeared: ‘Exhaustion’, ‘Continuity’ and ‘Planning and cooperation’. Although ‘Exhaustion’ appeared as a new factor, this aspect of care has been present as items in the MT dimension of the previous QPP instrument (personal communications with the author Wilde-Larsson). Previous research supports continuity, and planning and cooperation are important for patients who receive palliative care [32, 41, 42] and should be included in measures in palliative care [12]. Compared to existing instruments measuring palliative care quality from patients’ perspectives [18–22], QPP-PC consists of similar aspects of care. However, to our knowledge, previous instruments do not cover all these aspects of care in one single instrument.

Two items correlated with factors that differed from the original version of the QPP. The item about waiting times was previously described as a factor belonging to the MT dimension; however, in this study this item correlated with items in the factor ‘Access to help, food and equipment’, placed in the PT dimension. The item about individualized care was previously described as a factor belonging to ‘Routines’. In this study, this item correlated with items in the factor ‘Continuity’. By developing new items and testing these in a new context, new constructs of items may appear that could partly explain the differences experienced in this study. With regard to continuity, it is reasonable to think that receiving help from the same doctors and nurses influences individualized care, and these items may therefore be expected to correlate. These findings need to be supported by further studies; we suggest that further validation of the instrument be obtained by confirmatory factor analysis.

Reliability [40] was assessed by Cronbach’s α, and values were >0.7 for most factors and dimensions on both the PR and the SI scales, indicating good internal consistency [37]. This is in line with previous studies using the QPP in other contexts [29]. However, the factor ‘Access to help, food and equipment’, in the PT dimension, and the factor ‘Continuity’ showed α values <0.7. This α value for the PT dimension is in line with previous studies using the QPP in the context of hospital care [27, 39]. The α values are sensitive to the number of items, and low numbers may lead to low α values, which could possibly explain the results [36]. The factors ‘Spiritual and existential’ and ‘Meaningfulness’ gained high α levels, which may be explained by the items within both of these factors being the same questions asked by different healthcare personnel.

The limitation in this study could be related to sample size. Based on previous studies using the QPP in related samples [30, 31, 43], an expected medium effect size was estimated, resulting in inclusion of a minimum of 300 participants to achieve a minimum 80 % power. However, it is difficult to recruit large samples from this specific patient population, because they are in the palliative phase of their illness. Even though the data collection period was extended by six months, the estimated sample size was not achieved. This was due to a combination of factors, e.g., several patients with cognitive impairment and some patients who died before the implementation of already scheduled interviews.

The recommended sample size needed to perform a stable and reliable factor solution varies from a minimum of 50 cases to a ratio of participants to variables of 20:1, with a desired ratio of 5:1 [37]. Others argue that the minimum sample size is not valid and useful and that stable factor solutions can be achieved with a sample size that is considered too small for the above-mentioned ‘rules of thumb’ [44]. To improve items and to reduce the number of items, the research group performed critical assessments of all items on two occasions (after the pilot and before the PCA); this could enhance a stable factor solution. In this study, the KMO test was >0.8 for the whole instrument and >0.6 for all individual factors. In addition, a stable pattern of factors was present at the SI and PR scale levels and dimensional levels. Consequently, the sample size appeared to be sufficient to achieve a reliable and stable factor solution.

The advantages of an instrument developed for patients with different illnesses who get help from different services are many, i.e. when comparing groups of patients and services. However, the disadvantage is naturally higher proportions of the response alternative ‘not applicable’. For example, items about relief of shortness of breath may be regarded as highly relevant for some patients, on the other hand, irrelevant by patients with no difficulty breathing. Factors and dimensions in the QPP are computed by calculating a mean of all the items in the respective factors and dimensions. When patients respond ‘not applicable’ for one item in one factor or dimension, it is not possible to automatically calculate a factor or dimension score for this patient. For the T-tests, a mean therefor was calculated based on patients’ individual responses to the remaining items in the respective factor or dimension. For the PCA, a mean of the remaining responses in the respective variable substituted the “not applicable” response [38]. This allowed patients to be included in the analysis even when some items were recorded as ‘not applicable’. One may say that this recognizes ‘not applicable’ as a valid response alternative. However, this could imply a risk for correlations with the substituted mean rather than with the remaining patients’ responses to the respective items in the PCA [45] . To reduce this risk, seven patients with particularly high proportions of “not applicable” or missing responses were excluded from the analysis. However, the factors that appeared as a result of the PCA analysis needs to be interpreted with caution and to be supported by further studies.

QPP-PC identifies issues known in the literature as being important aspects of palliative care, in addition patients scored the subjective importance to be high for most of the factors, dimensions and single items. This indicates that QPP-PC could be considered to be generalizable and relevant. However, patients receiving palliative care in Norway are cared for in settings that have not been included in this study, e.g. hospital wards and nursing homes not specialized in palliative care. Even if the QPP-PC items were derived from hospital settings, as well as hospices, nursing homes and home care [24, 25, 32], and had been supported by a recent review to identify the most important aspects of palliative care in hospitals [46], the QPP-PC would need to be tested in other contexts, cultures and countries to support these findings.

A final limitation is that no specific measurement was made of the time needed by patients to complete the instrument.

### Discussion of result about patients’ perceptions of quality of palliative care

Most factors and dimensions on the SI scale were scored as of high or highest importance. High scores were also obtained for most factors and dimensions of the actual care received (PR scale). Previous studies using the QPP in palliative care showed similar results [30, 31]. However, the results revealed that patients scored on SI scales higher than on PR scales with regard to ‘Symptom relief’, ‘Information’, ‘Participation’, ‘Continuity’ and ‘Planning and cooperation’, indicating that patients experienced these aspects of care as not as high as they may have wished for (areas for improvement). Previous studies confirm that improvement in these aspects of care is needed [41, 42, 47–51]. Further research is needed to investigate whether subgroups of patients differ with regard to their perceptions of care quality and whether there are differences in perceptions of care quality between patients in different palliative settings.

## Conclusion

The QPP-PC is based on a theoretical model of quality of care, and has its roots in patients’ perspectives; this gives patients undergoing palliative care a voice when measuring and evaluating the quality of care. The instrument was contextually adapted and psychometrically evaluated in a sample of Norwegian patients receiving palliative care. Although further validation of the QPP-PC is needed to support these findings, the results are promising. Patients evaluated most factors and dimensions on the SI scale as of high or highest importance. High scores were also obtained for most factors and dimensions of the actual care received. Areas for improvement have been presented with regard to symptom relief, participation, continuity, and planning and cooperation.

### Clinical implications

The advantage of the QPP-PC is that the instrument includes measures of both subjective importance and perceived reality of care. This is particularly valuable for guiding the improvement of palliative care at all levels. Future improvement initiatives should focus on aspects of care receiving high scores on the SI scale and low scores on the PR scale (actual care received).

## Abbreviations

QPP: The instrument Quality from the Patient’s Perspective
QPP-PC: QPP instrument specific to palliative care
MT: Medical–technical competence dimension
ID: Identity-oriented approach dimension
PT: Physical–technical conditions dimension
SC: Sociocultural atmosphere dimension
SI: Subjective importance scale
PR: Perceived reality scale
EFA: Exploratory factor analysis
PCA: Principal component analysis
KMO: Kaiser–Meyer–Olkin test
